# LV5plex: Immune-histological phenotypes staged by self-studying for a liver cancer multiplex staining set

**DOI:** 10.3389/fcell.2023.1058987

**Published:** 2023-02-06

**Authors:** Dongbo Jiang, Xvshen Ding, Junqi Zhang, Yang Liu, Xiyang Zhang, Jijin Li, Jianing Shen, Yahui Shi, Yuancai Feng, Xupeng Qiao, Hengzheng Wei, Tengfei Zhuang, Yuanjie Sun, Shuya Yang, Fenli Zhou, Qingtao Zhao, Kun Yang

**Affiliations:** ^1^ Department of Immunology, Basic Medicine School, Air-Force Medical University (The Fourth Military Medical University), Xi’an, China; ^2^ Shaanxi Provincial Center for Disease Control and Prevention, Institute of AIDS Prevention and Control, Xi’an, Shaanxi, China; ^3^ College of Medical Technology, Shaanxi University of Chinese Medicine, Xianyang, Shaanxi, China; ^4^ State Key Laboratory of Cancer Biology, Xijing Hospital of Digestive Diseases, Fourth Military Medical University, Xi’an, Shaanxi, China; ^5^ Department of Pediatrics, Bethune International Peace Hospital of Chinese PLA, Shijiazhuang, Hebei, China

**Keywords:** tumour biomarkers, prognostic markers, liver cancer, fluorescence spectroscopy, machine learning

## Introduction

Primary liver cancer is the most frequent fatal malignancy (Anwanwan et al., 2020) and the sixth most common site of primary cancer in humans (Li et al., 2021). Primary liver cancer comprises hepatocellular carcinoma (LIHC), intrahepatic cholangiocarcinoma (CHOL), and other rare tumors, notably fibrolamellar carcinoma and hepatoblastoma (Sia et al., 2017). HCC and ICCA account for more than 90% of malignancies in the liver (Satriano et al., 2019). A variety of risk factors for liver cancer development have been identified, such as cirrhosis (chronic liver damage caused by fibrosis), hepatitis B virus (HBV) infection, hepatitis C virus (HCV) infection, alcohol abuse and aflatoxin B1 (European Association For The Study Of The Liver, 2012). The cancer has different pathological features and metabolic landscapes in different stages, so liver cancer can be diagnosed with the help of serologic testing, diagnostic imaging and histology (Grandhi et al., 2016), which are the basis for the traditional diagnosis of liver cancer stages (Xie, Ren, Zhou, Fan, Gao). Many staging systems are used in liver cancer staging, including the Tumor-Node-Metastasis (TNM)-based staging system, the Barcelona Clinic Liver Cancer (BCLC) staging system (1999 version), the American Joint Committee on Cancer (AJCC) staging system (8th edition, 2017) and the China liver cancer staging (CNLC) system (2018 version) (Amin et al., 2017; Piñeros et al., 2019).

However, most primary liver cancer patients are diagnosed at an advanced stage. At this stage, patients need to receive systemic therapy rather than surgical treatment (Villanueva, 2019). Thus, the application of a novel cancer staging method is significant when diagnosing and evaluating liver cancer patients. The ability of automated methods combined with morphological or immunohistochemical biomarkers has been demonstrated in the field of cancer diagnosis and treatment (Beck et al., 2011; Kather et al., 2019). This method is worth widely popularizing to replace classification methods, which are typically costly and labor intensive. A critical requirement for the development of these methods is the construction of datasets containing digitally scanned slides stained to show cell morphology and expression of regioselective proteins with accompanying staging outcomes.

B-cell receptor-associated protein 31 (BAP31) was named for copurification with the B-cell receptor component (Kim et al., 1994). It may also be noted that, relative to the corresponding non-cancerous tissues, BAP31 is generally highly upregulated in cancer, and it is expressed by many commonly used cell lines (such as HeLa, HEK293, CHO, COS-7 and BHK-21) (Lambert et al., 2001; Wakana et al., 2008; Wang et al., 2008). BAP31 has the potential to be used as a prognostic marker for several different forms of cancer (Yu et al., 2015; Tan et al., 2016; Wang et al., 2020). BAP31 can also bind and upregulate serpin family E member 2 (SERPINE2), resulting in an increase in the phosphorylation levels of Erk and p38. Western blot showed that the expression levels of Erk1/2, phospho-Erk1/2, and phospho-p38 were significantly decreased when SERPINE2 was knocked down; however, the BAP31 expression was not different in these cells. Inhibition of SERPINE2 attenuated BAP31-induced cell proliferation (Zhang et al., 2020). These phenomenon suggest that SERPINE2 is a downstream gene of BAP31 and may regulate cell proliferation by influencing the phosphorylation of Erk1/2 and p38. Lipoprotein receptor-related protein-1 (LRP-1), the downstream gene of SERPINE2, is also positively correlated with SERPINE2 in human tumors (Fayard et al., 2009). Through limited prognostic assessment in stage I or II breast cancer, Ki-67 immunohistochemistry (IHC) is commonly used as a tumor proliferation marker and plays a significant role (Nielsen et al., 2021). The expression levels of LRP1 and Ki-67 also showed a positive correlation with the other two biomarkers. The regulatory relationship among these four biomarkers is specific to primary liver cancer. Thus, the thought arises of the relevant four biomarkers which could assist the diagnosis of primary liver cancer.

Here, we show our dataset, which contains two digitally scanned high-resolution tissue microarrays (TMAs) named LV1221 and LV 2089, which comprise tissue core sections from a total of 303 samples. Both TMAs were stained for HE. Some specific biomarkers (BAP31, SERPINE2, Ki-67, LRP1) and DAPI (4,6-diamidino-2-phenylindole) were also stained. Each core was provided with clinical data, pathologist annotation, and staging information. We segmented cells from tissue and computed the expression level of biomarkers in cancer cells by inForm advanced image analysis software (inForm 2.4.1, PerkinElmer). The exported outputs constitute part of the dataset. The utility of our dataset was proven by performing statistical analysis. Its application can avoid a massive workload in related laboratories and provide better guidance for the diagnosis of primary liver cancer.

## Materials and methods

We first performed a panoramic scan of the primary liver cancer tissue microarrays LV1221 and LV2089 by Aperio GT 450 (Leica Biosystems, United States). All samples were annotated, and a unique identifier was numbered. Examples of relevant images are shown in the Supplementary Material. Slides were then stained for the specific biomarkers by multiplex immunohistochemistry (mIHC) technology. The advantage of mIHC staining is that it allows simultaneous detection of multiple biomarkers on a single tissue section and the comprehensive study of cell composition, cellular functional and cell-cell interactions *in situ* (Tan et al., 2020). The stained slides were scanned using the PerkinElmer Mantra System (PerkinElmer, United States) to obtain IM3 format pictures. The IM3 format pictures were then unmixed by inForm advanced image analysis software (inForm 2.4.1, PerkinElmer) to prepare for subsequent quantitative analysis. Representative cores were selected to train the inForm and manually set parameters so that the inForm could realize automated and massive analysis. This analysis process consists of three steps, including tissue segmentation, cell segmentation and phenotype. In the phenotype step, we separated cancer cells and calculated the expression levels of four markers. The results were recorded as the H-score, which can help us quantitatively analyze the influence of these biomarkers on the progression of primary liver cancer.

### Patient cohort

The study cohort consisted of 303 primary liver cancer patients (age 27–77), including 94 women and 209 men. We acquired 331 tissue samples from them and stored them in LV1221 and LV 2089.

### Ethic statement

This study was approved by the institutional review board of the Fourth Military Medical University. Our samples are commercial TMAs, and the supplier has completed the informed consent of all patients in the process of data collection.

### HE and pathologist annotations

The HE-stained original primary liver cancer tissue microarrays were scanned by an Aperio GT 450 (Leica Biosystems, United States) to obtain SVS format pictures, and TIF format pictures were also exported for convenient visualization. Due to large pieces of defect and non-tumor cell regions, these samples were independently evaluated and diagnosed by two or more pathologists, and inappropriate parts were excluded. We acquired 309 available cores and annotated them with diagnoses, grade, TNM, stage, type, basic information of patients and a unique identifier.

### Multiplex immunohistochemistry staining and multispectral imaging

**FIGURE 1:**
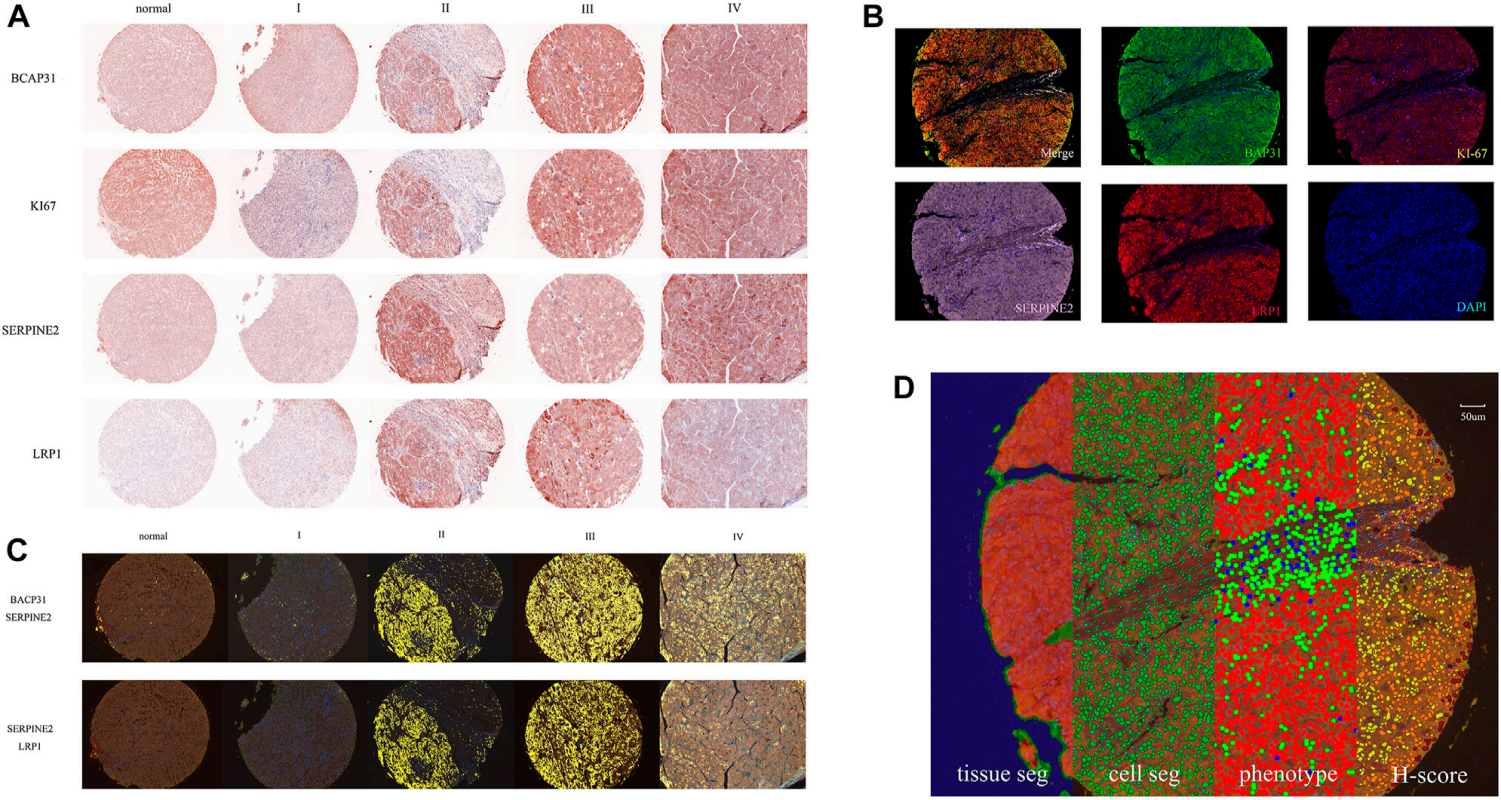
The whole process of data and image acquisition: **(A)** The expression levels of four biomarkers in different stages were determined by multiplex immunohistochemistry staining technology. The darker color indicates that the biomarker expression showed an upward trend. **(B)** A stained core was multispectral unmixed, the merged image was unmixed into five pictures: Opal520-stainig BAP31 (green), Opal570-stainig Ki-67 (brown), Opal620-stainig SERPINE2 (pink), Opal690-stainig LRP1 (red) and DAPI (blue). **(C)** Colocalization of BAP31 and SERPINE2. The colocalization analysis was performed. The percentage of colocalization area (golden) was calculated. **(D)** All three steps to train the inForm for segmentation and the calculation of the H-score. From left to right are the tissue segmentation, cell segmentation, phenotype and H-score. Each step is a further analysis based on the previous step. Different tissues were first divided into cancer (red), stromal (green), and background (blue) tissues. Then, the cells were segmented. Finally, the software-trained algorithm recognized cancer cells (red), immune cells (green), and stromal cells (blue). According to the biomarker expression level, we set thresholds to divide cells into 4 grades (blue means no expression, yellow means +1 expression, orange means +2 and brown means +3) and calculated the H-score.

The original slides of TMAs LV2089 and LV1221 were processed through 5-μm-thick sections, deparaffinized in xylene, and rehydrated in an ethanol gradient. Slides were stained according to Opal 7-plex technology (PerkinElmer) so that we could simultaneously visualize four markers and DAPI (4,6-diamidino-2-phenylindole) for nuclei on the same slide. During each of the six cycles of staining, antigen retrieval (AR) was conducted *via* microwave treatment in AR solution pH 6 or pH 9 (AR6 or AR9) suggested by IHC validation; blocking was followed by incubation for 15 min at room temperature (RT); and primary antibodies [anti-BAP31 mouse monoclonal antibody, anti-SERPINE2 antibody, anti-LRP1 antibody (Abcam; ab92544), and anti-Ki67 antibody (Immunoway; YM6189)] were then incubated for 1 h at RT or overnight at 4°C. Next, HRP labeled polymer goat anti-mouse and rabbit antibodies were incubated at RT for 10 min followed by TSA opal fluoroscopes (Opal 520, Opal 570, Opal 620, and Opal 690) for 10 min, which indicated BAP31, Ki-67, SERPINE2, LRP1, respectively. Microwave treatment was performed to remove the antibody TSA complex at each cycle of staining with AR solution (pH 9 or pH 6) (Tan et al., 2020; Govek et al., 2021). Finally, all 303 available samples were counterstained with DAPI for 5 min and mounted in ProLong Antifade Mountant (Solarbio). The slides were scanned using the PerkinElmer Mantra System (PerkinElmer, United States) to acquire multispectral images (Zhang et al., 2020). An example of these images is shown in Figure 1A.

### Multi-spectrum unmixing

The multispectral images were unmixed by the inForm Advanced Image Analysis software (inForm 2.4.1 PerkinElmer), we chose NJGL OPAL7C as Select Fluors and distinguished Opal 520, Opal 570, Opal 620, Opal 690, and DAPI as green, brown, pink, red, and blue. Example unmixed pictures are shown in Figure 1B. Except for exported pictures for single biomarkers, we also conducted colocalization analysis for pixels after unmixing and found correlations among these biomarkers. Examples of BAP31_SERPINE2 colocalization and SERPINE2_LRP1 colocalization are shown in Figure 1C. We set a coordinate axis to describe the location of each pixel and used pixels to calculate the region area (square microns), region position and proportion. These processes could help us understand their interaction in tumor growth.

### Machine learning

To score the biomarker expression level on cancer cells, multispectral images representative of different samples were selected and used to train the inForm software for segmentation. The scoring protocol consisted of three automated steps, including tissue segmentation, cell segmentation and phenotype. In the tissue segmentation, the inForm software was trained using specific samples from each category to automatically segment each image into the cancer region (red), stromal region (green), and background (blue). The parameter including the minimum segment size is 500 pixels, and the edges are trimmed by 5 pixels. Images from all samples were segmented, reviewed, merged and exported for further analysis using the software’s batch processing and merge functions, and these exported data were then merged into a single data set for analysis. The percentage area of different tissues was also determined. In the cell segmentation, based on the nuclear dye (DAPI), we set the minimum nuclear size as 25 nm to segment each cell from tissue. In the phenotype, the algorithm was developed to segment cancer cells (red), immune cells (green), and stromal cells (blue) by training the software on a limited number of regions. An example of all three steps is shown in Figure 1D. Cells in the tissue were then phenotyped and counted by training the inForm software to recognize each cell type. After segmenting the cancer cells, we selected a few samples and artificially set 3 thresholds to divide the samples into 4 grades (blue means no expression, yellow means +1 expression, orange means +2 and brown means +3) according to the biomarker expression level. An example of BAP31 is shown in Figure 1D. By calculating the weighted average between grades and proportions of cells with different expression levels, we obtained the H-score to quantify the expression of each biomarker.

## Data records

**FIGURE 2:**
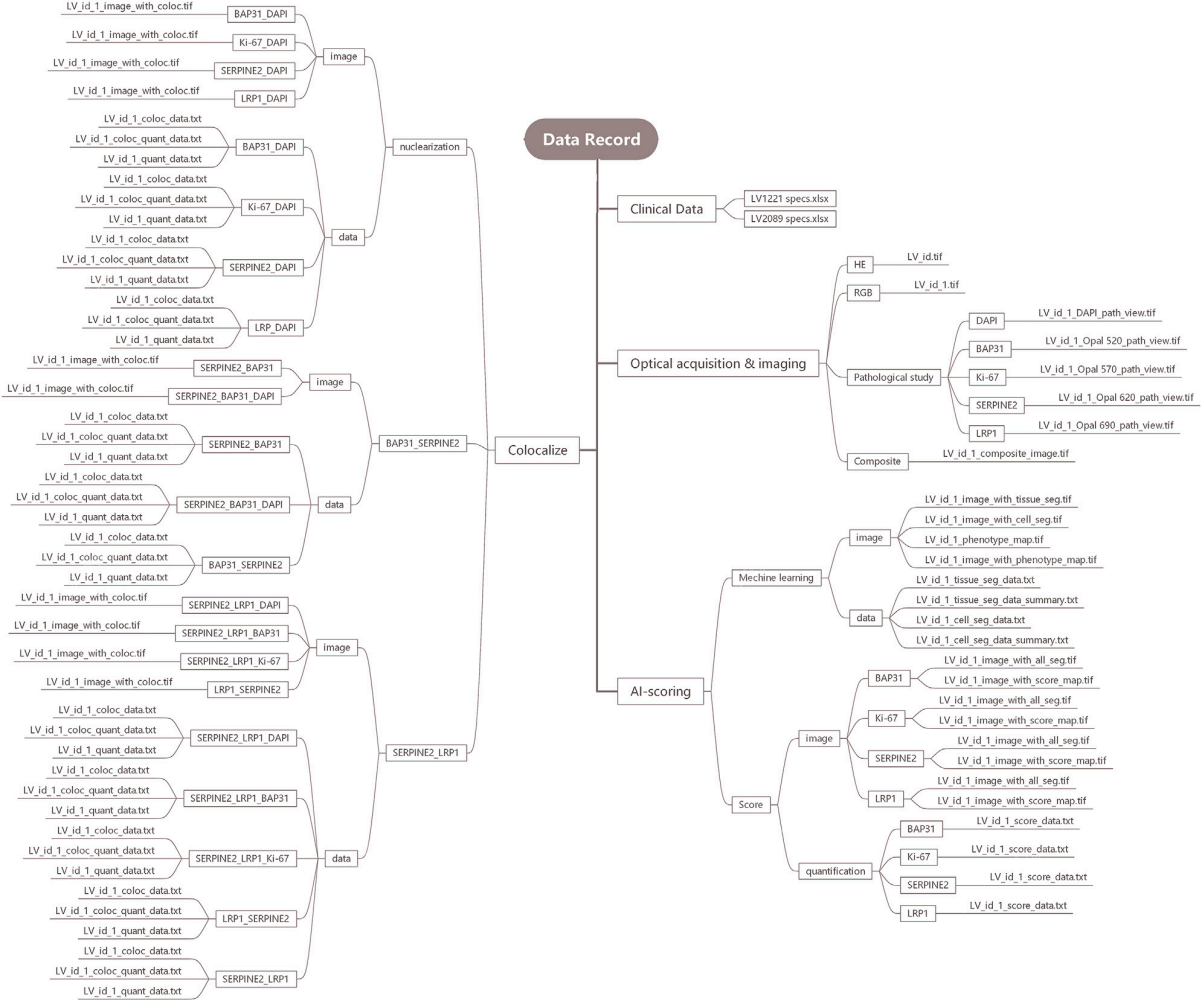
Detailed structure drawing of the data record.

We processed the cores from tissue microarrays LV2089 and LV1221 by inForm v2.4.1 and built the data records. The data records are organized into four folders, including Clinical data, Optical acquisition and imaging, Machine Learning (artificial intelligence, AI) based ROI scoring, and Colocalize. The structure of our data records is shown in Figure 2.

### Clinical data

The clinical data of the patients are stored in LV1221 specs.xlsx and LV2089 specs.xlsx, which contain pathologist annotations, including age, sex, diagnosis, grade and unique identifiers for each sample.

### Optical acquisition and imaging

The Optical acquisition and imaging folder contains four parts: HE, RGB, Pathological Study, and Composite. They contain optical images of 309 available samples and are stored in TIF format. The HE part contains the HE-stained original pathological sections of all 309 samples. The RGB part contains RGB pictures from these available samples. The composite part contains 309 composite images to analyze the expression levels of four markers in all available samples, and BAP31, Ki-67, SERPINE2, LRP1, and DAPI were distinguished as green, brown, pink, red, and blue by IHC staining technology. The Pathological Study part contains pathological view for BAP31, Ki-67, SERPINE2, LRP1, and DAPI.

### AI-scoring

The AI-scoring folder contains two parts, Machine Learning and Score. In the Machine Learning part, we analyzed samples in three steps: tissue, cell and phenotype. In the tissue segmentation step, the cancer region, stroma region and background were stained red, green, and blue, respectively. In the cell segmentation step, all cell nuclei larger than 25 nm were identified and stained, and in the phenotype step, the cancer, immune and stromal cells were distinguished as red, green and blue. We organized these outputs into two subfolders, image and data. The image folder includes tissue_seg.tif, cell_seg_map.tif, phenotype_seg_map.tif and phenotype_map.tif. The data folder includes tissue_seg_data.txt, tissue_seg_data_summary.txt, cell_seg_data.txt, and cell_seg_data_summary.txt, and the phenotype data are included in the cell section. In the Score part, the expression levels of four markers in the cancer cells were calculated, and the results were expressed as the H-score. We organized the outputs into two subfolders, image and quantification, where the former contains all_seg.tif and score_map.tif and the latter contains score_data.txt.

### Colocalize

The Colocalize folder contains three parts: nuclearization, BAP31_SERPINE2 and SERPINE2_LRP1. In the nuclearization part, it contains colocalization analysis outputs of each marker with DAPI. In the BAP31_SERPINE2 and SERPINE2_LRP1 parts, to further understand the influence of the colocalization relation on cancer, the biomarker colocalization relation was analyzed, and the outputs were divided into the following parts: BAP31_SERPINE2 and SERPINE2_LRP1, where the former contains BAP31_SERPINE2 (analyzing BAP31 based on SERPINE2), SERPINE2_BAP31 (analyzing SERPINE2 based on BAP31), and the latter contains SERPINE2_LRP1 (analyzing SERPINE2 based on LRP1), LRP1_SERPINE2 (analyzing LRP1 based on SERPINE2). We calculated the colocalization region area and proportion. The outputs, including coloc_data.txt, coloc_quant_data.txt, and quant_data.txt, were stored in the data subfolders, and coloc.tif was stored in image subfolders.

## Technical validation and result

Taking the example of BAP31 and SERPINE2, we used the H-score and percentage of the colocalized area to test the usability of these data in our dataset. This analysis was performed on 16 cores from normal liver tissues, 5 cores from cancer adjacent liver tissues, and 250 cores from primary liver cancer tissues, including intrahepatic cholangiocarcinoma (CHOL) and hepatocellular liver cancer (LIHC). All analyses were performed in GraphPad Prism software 7.0 (SanDiego, CA, United States). The methods used included one-way analysis of variance (ANOVA) and the chi-square test.

ANOVA was used to compare the mean H-score in different groups and stages (McHugh, 2011). We set three groups, cancer, normal + adjacent and normal, to compare the means and obtained a *p*-value of 0.0059 (*p* < 0.05 was considered statistically significant). After performing the same analysis for SERPINE2, the result was also statistically significant (*p* < 0.0001). The above results indicated that the expression of these two biomarkers is discrepant in different tissues, and this conclusion matches the result in GEPIA2 (http://gepia2.cancer-pku.cn/#index) (Tang et al., 2019). We also used ANOVA to compare the expression of BAP31 in different stages. The H-score was used to represent the expression level. We performed ANOVA on BAP31 expression levels in different stages and obtained a *p*-value of 0.0004. This result indicated that the expression of BAP31 varies in different stages. To further understand this difference, we also performed a chi-square test. To realize the chi-square test analysis of H-score and stages, H-score was converted from continuous variable to categorical variable by dividing it into two parts based on the median, above the median and below the median were represented by 1 and 0 respectively, stage I, II and III, IV were represented by Early and Advance respectively. Through software analysis, we also obtained a *p*-value equal to 0.0236, which indicated that BAP31 expression varies in different stages, and the graph showed an increasing trend (Dang et al., 2018). The percentage of the colocalized area was also analyzed by ANOVA. We selected the colocalization of SERPINE2 and BAP31 to perform ANOVA with four stages and obtained a *p*-value equal to 0.0065. Colocalization showed the percentage of the colocalized area of the two biomarkers in the whole cores, and its size may be related to the degree of interaction between the two biomarkers. This result could indicate that the interaction of the two biomarkers would increase with the development of cancer. Moreover, through the signature query function of GEPIA2, the overall survival time of the high 2 signatures (BAP31 and SERPINE2) group and the low 2 signatures (BAP31 and SERPINE2) group showed significant differences (*p* < 0.05). It may also be noted that, the survival information of liver cancer patients from another database TCGA (https://www.cancer.gov/about-nci/organization/ccg/research/structural-genomics/tcga) shows a trend, which is that the overall survival (months) shortens with the increased transcription of the corresponding mRNA (Blum et al., 2018). This result validates the usability of our database for survival assessment. Our results suggest that data from multispectral analysis can provide an important effect on liver cancer diagnosis and should be further studied on the basis of a larger patient cohort. The supplementation of survival outcomes can also provide a vital signal to predict survival outcomes (Vrabac et al., 2021).

### Usage notes

The structure of the data record is shown in Figure 2. The data files from the inForm software can be easily merged by Macros in Excel and further processed by GraphPad Prism 7. We selected GEPIA2 (http://gepia2.cancer-pku.cn/#index) as an external dataset to verify the validity of the data. However, compared with external datasets, the data have no survival outcomes, so further study of our data can focus on developing the ability to predict the survival of patients. All data records are publicly available at: https://github.com/Xvshen/code-availability.

## Data Availability

The original contributions presented in the study are included in the article/Supplementary Material, further inquiries can be directed to the corresponding authors.
