# Supplementary material for: LV5plex: Immune-histological phenotypes staged by self-studying for a liver cancer multiplex staining set
Source: Front Cell Dev Biol. 2023 Feb 6;11:1058987. doi: 10.3389/fcell.2023.1058987 (PMC9940753; doi:10.3389/fcell.2023.1058987)
Supplement: Supplementary file 1 [file DataSheet1.pdf]

Supplementary Material

Supplementary Figures

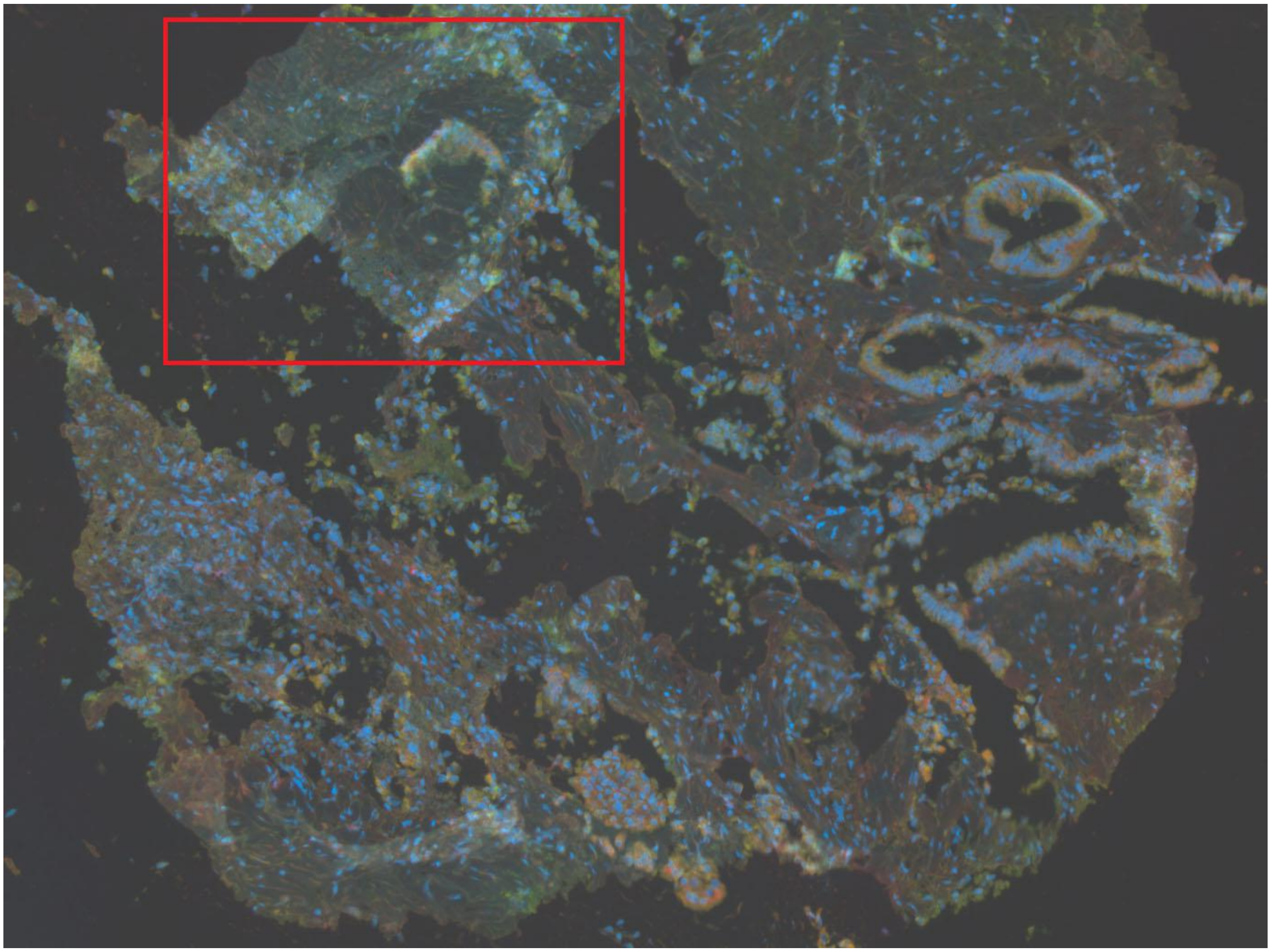

Supplementary Figure 1. The annotated example of picture excluded. A core from the TMA was artificially annotated to exclude the unrepresentative part by red rectangle.

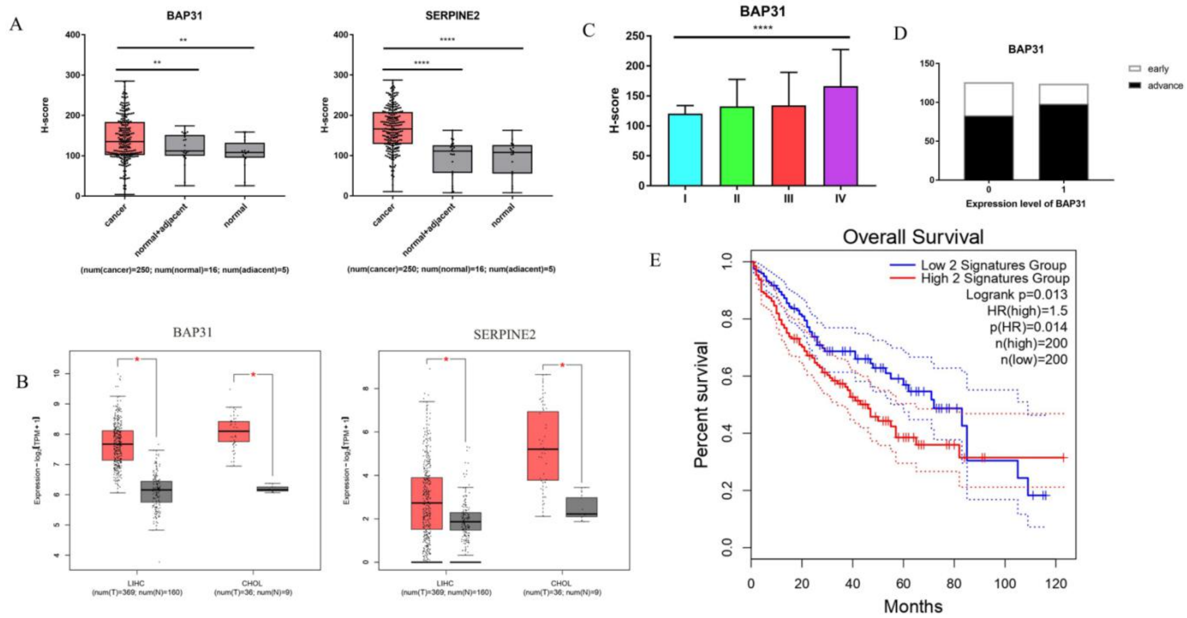

Supplementary Figure 2. The results are shown in graphs and compared with data from GEPIA2. **(A)** Images of biomarkers BAP31 and SERPINE2 expressed in different groups. **(B)** Boxplots acquired from GEPIA2. The LIHC and CHOL groups were both statistically significant when compared with the normal groups. **(C)** BAP31 expression is obviously different in the four stages. **(D)** The expression level of BAP31 was defined as 0 and 1, and a chi-square test was performed with processed stages (I, II represented by early, III, IV represented by advanced). **(E)** The statistical results of overall survival under the influence of two signatures (BAP31 and SERPINE2) in the joint cohort of LIHC and CHOL from TCGA by GEPIA2.

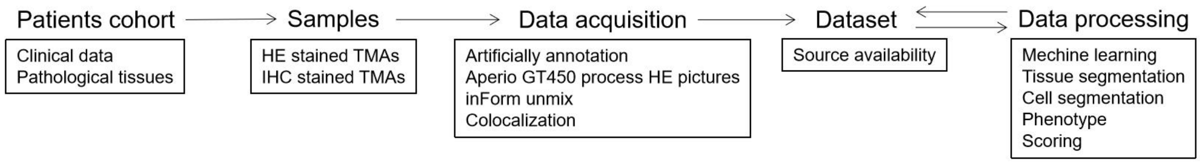

Supplementary Figure 3. A brief framework of LV5plex pipeline. The whole process is divided into five parts from case report to information acquisition. Each part is respectively annotated with a series of key steps displayed in the figure.
